# An assessment of medical students’ knowledge of prediabetes and diabetes prevention

**DOI:** 10.1186/s12909-019-1721-9

**Published:** 2019-07-29

**Authors:** Tamkeen Khan, Gregory D. Wozniak, Kate Kirley

**Affiliations:** Improving Health Outcome, American Medical Association, 330 N. Wabash Avenue, Suite 39300, Chicago, IL 60611 USA

**Keywords:** Prediabetes, Diabetes prevention, Physician training, Medical education, Educational measurement

## Abstract

**Background:**

The United States has 84 million adults with prediabetes, putting them at a higher risk than the general population for developing type 2 diabetes. Missed opportunities among primary care providers in diagnosing and managing patients with prediabetes represent a gap in care, suggesting there is a need to educate practicing physicians and medical students about diabetes prevention. The purpose of this study is to assess medical students’ basic knowledge of prediabetes and diabetes prevention, identify potential educational needs, and target areas for improvement in undergraduate medical education curricula.

**Methods:**

A cross-sectional study to assess medical students’ preclinical and clinical management knowledge of prediabetes and diabetes prevention. Medical students attending the 2016 American Medical Association’s annual meeting took a 6-item knowledge questionnaire using a mobile application or a paper version. Scores were reported for the full sample of respondents, by year in medical school, by topic area, and by mode of survey response.

**Results:**

The average student answered fewer than half of the questionnaire questions correctly. Scores on some items addressing preclinical content were higher among third- and fourth-year students compared to first- and second-year students (*p* = 0.039 and effect size = 0.363). Average scores on the items addressing clinical management were not significantly different by year in medical school, but the item measuring effectiveness of metformin to a lifestyle change program had 41.9% correct answers among the mobile application respondents compared to 21.5% among paper test respondents (*p* = 0.003 and effect size = 0.463).

**Conclusions:**

Medical student performance on the prediabetes knowledge questionnaire was low. Students’ year in medical school had a slight impact on overall performance, but only for certain questions. The results suggest the need for improvements in current medical school curricula for increasing the awareness of screening for prediabetes as well as the benefits of the lifestyle change programs in the National Diabetes Prevention Program.

**Electronic supplementary material:**

The online version of this article (10.1186/s12909-019-1721-9) contains supplementary material, which is available to authorized users.

## Background

Nearly 1 in 3 adults in the United States (US) has prediabetes, a condition characterized by blood glucose levels that are elevated- hemoglobin A1C (HbA1c) test results between 5.7 and 6.4%- but not high enough to be classified as diabetes [1]. Risk factors for prediabetes include family history of type 2 diabetes, history of gestational diabetes, elevated body mass index (BMI), and sedentary lifestyle. Individuals with prediabetes are at higher risk than the general population for developing type 2 diabetes, heart disease, stroke, and other serious health conditions [1]. People with prediabetes can reduce their risk of developing type 2 diabetes by participating in structured lifestyle change programs (LCPs). Many LCPs currently available are modeled after the original Diabetes Prevention Program research study focused on promoting healthy diet, weight loss and increased physical activity, and are recognized by the Centers for Disease Control and Prevention’s (CDC) National Diabetes Prevention Program (National DPP) [2, 3]. Research showed that participation in an LCP reduced the incidence of type 2 diabetes by 58% relative to placebo at an average follow-up time of 3 years. In comparison, those treated with metformin had a 31% reduced 3-year incidence of type 2 diabetes compared to the placebo group [3]. Primary care physicians play a key role in screening, testing, and referring patients with prediabetes to LCPs.

The evidence base for preventing diabetes via intensive lifestyle change is substantial [4–9]. The United States Preventive Services Task Force (USPSTF) incorporated this evidence into the updated recommendation regarding screening for abnormal glucose and type 2 diabetes [10]. The grade B recommendation states that physicians should screen individuals for abnormal glucose if they are between the ages of 40 and 70 and are overweight or obese. USPSTF also recommends that all individuals with abnormal glucose should be referred for intensive behavioral counseling to promote lifestyle change [11]. Despite the evidence supporting intensive LCPs and clinical guidelines encouraging physicians to refer patients to these programs, they are still vastly underutilized and providers’ awareness and patient referral rates to LCPs are still low [12–14].

Attitudes towards prediabetes coupled with missed opportunities among these providers in diagnosing and managing prediabetes represent a practice gap [15]. A recent survey determined that primary care physicians have significant knowledge gaps regarding prediabetes screening, diagnosis, and management, with less than 20% of physicians correctly answering questions in those domains [16]. These practice and knowledge gaps are an area where the American Medical Association (AMA) is focusing efforts towards educating physicians to screen and refer individuals with prediabetes to CDC-recognized LCPs [17]. Given that the practice and knowledge gaps observed by both Mainous et al. and Tseng et al. were so substantial, this begs the question of whether physicians in-training are receiving adequate prediabetes-related education [15, 16].

Limited information is available on whether prediabetes management or diabetes prevention is being taught in undergraduate medical education (UME) curricula. Physicians in training lack confidence in management of diabetes, report a need for further training [18], and often benefit from additional educational interventions and resources [19]. Furthermore, there is no information publicly-available from the National Board of Medical Examiners that describes whether medical students are being assessed on prediabetes or prevention of type 2 diabetes, specifically in the United States Medical Licensing Examination. This study attempts to measure medical students’ knowledge of prediabetes and diabetes prevention to identify the potential educational needs and areas for improvement in chronic disease prevention curricula in UME.

## Methods

A 6-item multiple choice questionnaire (Additional file 1) was developed to assess medical students’ basic knowledge of prediabetes and type 2 diabetes prevention. The items were designed to reflect common domains typically used to teach disease-specific knowledge to medical students: epidemiology, diagnosis, and management, which included treatment options and clinical guidelines. These survey topic areas align to the key knowledge domains for prediabetes.

We relied on 3 subject-matter experts, including 2 primary care physicians to assess the questionnaire. Their review indicated the knowledge domains were addressed by the items with an appropriate level of difficulty for medical students.

The questionnaire was administered to a convenience sample of medical students attending the AMA’s House of Delegates (HOD) meeting on June 11–15, 2016 in Chicago, Illinois during the Medical Student Section (MSS) sessions. To minimize the burden of response the only demographic characteristic collected from the respondents was year in medical school. Respondents entered data into the AMA meeting mobile application (Crowd Compass, Inc.) or a paper form. Participants were provided with an AMA gift bag as an incentive for completing the questionnaire. The University of Illinois Office for the Protection of Research Subjects Institutional Review Board approved the research protocol.

The percentage of participants correctly answering the individual items and average total scores are reported for the full sample and by the two groups of students, first- and second-year students, and third- and fourth-year students. The questions were further broken into two categories defined according to the timing in which medical students might typically be taught each item: in the preclinical years or clinical years. Preclinical content was focused on epidemiology and diagnosis (proportion of adults with prediabetes, risk factors for prediabetes, and HbA1c levels), while clinical content was focused on the management of prediabetes (USPSTF screening recommendations, abilities of the LCP and metformin to reduce incidence of diabetes, and recommendations for clinically significant weight loss ranges). To explore whether lower performance on specific questions might be explained by timing of exposure to specific content in their curriculum, we assessed differences in performance on preclinical and clinical questions by year in medical school. Differences in scores between years of medical education and instrument modality (mobile application versus paper format) were examined using analysis of variance (ANOVA). Statistical significance is presented at both *p* < 0.05 and *p* < 0.10 since significance levels are a decreasing function of sample size [20, 21]. Effect sizes were also calculated, with an effect size greater than 0.33 used to distinguish differences of practical significance between the means [22]. All analyses were performed in STATA 13 (College Station, TX).

## Results

There were 600 medical students who attended the meeting; 258 respondents completed the questionnaire; 61 respondents were residents, fellows, or attending physicians who wished to test their knowledge; the remaining 197 current medical students were used in the analysis. The medical students who attended the AMA HOD meeting, represented a total of 138 schools. Among the 197 current medical students, over three-quarters (*n* = 156) were first- and second-year students, and the remaining sample (*n* = 41) were third- and fourth-year students.

The question items and response frequencies are shown in Table 1. Among all respondents, almost 60% correctly answered the question about the optimal weight loss range for preventing or delaying the onset of type 2 diabetes. On the other hand, only 13% responded correctly to the question about the USPSTF recommendations for screening. Roughly half the respondents answered questions about prediabetes prevalence and risk factors correctly, and slightly more than a quarter of the respondents correctly answered questions about prediabetes diagnosis and interventions to prevent diabetes.

**Table 1:** Knowledge Questions and Response Frequencies

*Preclinical knowledge of prediabetes and diabetes prevention*	Response (%)
Q1. What proportion of adults in the U.S. has prediabetes?	
a. 1 in 2	11.17
b*. 1 in 3*^*a*^	*48.22*
c. 1 in 5	36.04
d. 1 in 10	4.57
Q2. Which of the following is NOT a risk factor for prediabetes	
a. Family history of type 2 diabetes mellitus	2.54
b. History of gestational diabetes	5.58
c. *BMI of 20*^a^	*53.30*
d. Asian race	38.07
e. No answer	0.51
Q3. A patient with an HbA1c of 6.5 has prediabetes?	
a. True	73.60
b*. False*^*a*^	*26.40*
*Clinical management knowledge of prediabetes and diabetes prevention*	Response (%)
Q4. The 2015 USPSTF recommendation statement about screening for abnormal glucose and type 2 diabetes mellitus recommends screening adults for abnormal glucose if they are:	
a. Overweight and obese at any age	44.16
b*. Age 40 to 70 and overweight or obese*^*a*^	*12.69*
c. Over the age of 45	10.66
d. Over the age of 45 and have at least one additional risk factor for abnormal glucose	31.98
e. No answer	0.51
Q5. Which of the following best describes the abilities of metformin and the National DPP [LCP] to reduce the incidence of type 2 diabetes mellitus among individuals with prediabetes?	
a. Neither metformin nor the National Diabetes Prevention Program are more effective than placebo	2.54
b. Both metformin and the National Diabetes Prevention Program are more effective than placebo, and they are similarly effective to each other	53.81
c. Metformin is nearly twice as effective as the National Diabetes Prevention Program (both are better than placebo)	15.23
d. *The National Diabetes Prevention Program is nearly twice as effective as metformin (both are better than placebo)* ^*a*^	*27.92*
e. No answer	0.5
Q6. Individuals who participate in the National Diabetes Prevention Program [LCP] can help prevent or delay the onset of type 2 diabetes if they lose a minimum of:	
a. *5 to 7% of their body weight*^*a*^	*58.88*
b. 10 to 12% of their body weight	36.55
c. 15% of their body weight	4.57

^a^Indicates the correct answer

The percentage of correct responses by item and year in medical school are shown in Table 2. When separated by years in medical school, almost 40% of the third- and fourth-year students correctly responded to the question about HbA1c levels, while fewer than one-quarter of the first- and second-year students answered correctly (*p* = 0.039 and effect size = 0.363). Table 3 shows that the overall mean scores for the preclinical items were higher than the scores for the clinical management questions. Although some preclinical items demonstrated a trend towards better performance for third- and fourth-year students, their scores were not statistically different from first- and second-year students. Mode of response scores are shown in Table 4, where almost 60% of the students who took the test on paper responded correctly to the preclinical question regarding prediabetes risk factors, compared to 40% of electronic mobile respondents (*p* = 0.013 and effect size = 0.385). Interestingly slightly over 40% of electronic respondents correctly answered the clinical item comparing the effectiveness of metformin versus LCP, relative to a little over 20% of the paper respondents (*p* = 0.003 and effect size = 0.463). Clinical management scores were lower for those who took the test on paper (*p* = 0.048) compared to those who took the test using the mobile application, however the effect size was small at 0.078 as shown in Table 5.

**Table 2:** Percentage of Participants Correctly Answering Prediabetes and Diabetes Prevention Knowledge Questions, by Year in Medical School

	All Students (*n* = 197)	1st & 2nd year Students (*n* = 156)	3rd & 4th year Students (*n* = 41)	*p*-value [effect size]
*Preclinical knowledge of prediabetes and diabetes prevention*				
Q1. Proportion of adults with prediabetes	48.22	50.00	41.46	0.333 [0.170]
Q2. Prediabetes risk factors	53.30	50.00	65.85	0.071* [0.320]
Q3. HbA1c levels	26.4	23.08	39.02	0.039** [0.363]
*Clinical management knowledge of prediabetes and diabetes prevention*				
Q4. USPSTF recommendations	12.69	12.18	14.63	0.677 [0.072]
Q5. Metformin vs LCP	27.92	27.56	29.27	0.830 [0.038]
Q6. National DPP weight loss	58.88	59.62	56.10	0.686 [0.071]

*p*-values student scores are significantly different from 1st & 2nd year students compared to 3rd & 4th year students; * *p* < 0.10; **p < 0.05;

significant effect size for educational research is ≥0.33

**Table 3:** Mean Preclinical and Clinical Scores by Year in Medical School (Maximum Score 3.0)

	All Students (*n* = 197)	1st & 2nd year Students (*n* = 156)	3rd & 4th year Students (*n* = 41)	*p*-value [effect size]
Preclinical knowledge of prediabetes and diabetes prevention	1.28	1.23	1.46	0.114 [0.275]
Clinical management knowledge of prediabetes and diabetes prevention	0.99	0.99	1.00	0.960 [0.014]

*p*-values student scores are significantly different from 1st & 2nd year students compared to 3rd & 4th year students; significant effect size for educational research is ≥0.33

**Table 4:** Percentage of Participants Correctly Answering Prediabetes and Diabetes Prevention Knowledge Questions, by Mode of Response

	All Students (*n* = 197)	Paper (*n* = 135)	Electronic (*n* = 62)	*p*-value [effect size]
*Preclinical knowledge of prediabetes and diabetes prevention*				
Q1. Proportion of adults with prediabetes	48.22	48.15	48.39	0.975 [0.006]
Q2. Prediabetes risk factors	53.30	59.26	40.32	0.013** [0.385]
Q3. HbA1c levels	26.4	25.19	29.03	0.572 [0.086]
*Clinical management knowledge of prediabetes and diabetes prevention*				
Q4. USPSTF recommendations	12.69	14.07	9.68	0.392 [0.132]
Q5. Metformin vs LCP	27.92	21.48	41.94	0.003*** [0.463]
Q6. National DPP weight loss	58.88	57.04	62.90	0.440 [0.119]

*p*-values student scores are significantly different from students who took the test electronically compared to students who took the test on paper; **p < 0.05; ***p,0.01; significant effect size for educational research is ≥0.33

**Table 5:** Mean Preclinical and Clinical Scores by by Mode of Response (Maximum Score 3.0)

	All Students (*n* = 197)	Paper (*n* = 135)	Electronic (*n* = 62)	*p*-value [effect size]
Preclinical knowledge of prediabetes and diabetes prevention	1.28	1.18	1.33	0.249 [0.209]
Clinical management knowledge of prediabetes and diabetes prevention	0.99	0.93	1.15	0.048** [0.178]

*p*-values student scores are significantly different from students who took the test electronically compared to students who took the test on paper; **p < 0.05; significant effect size for educational research is ≥0.33

## Discussion

The results suggest that medical students’ overall knowledge of preclinical and clinical management of prediabetes and diabetes prevention was poor. The average student respondent failed to answer more than one-half of the questions correctly. The questions with the lowest scores, where less than one-third of the respondents answered correctly, were related to knowledge of USPSTF recommendations, HbA1c levels for prediabetes diagnosis, and the effectiveness of metformin and the LCP to reduce the incidence of type 2 diabetes. The year in medical school had a relatively small impact on overall performance, affecting only the questions regarding HbA1c levels and prediabetes risk factors for first- and second-year students versus those in their third- and fourth-year.

Prediabetes knowledge and practice gaps that exist among practicing physicians might be partially explained by inadequate training received by medical students. Inclusion of prediabetes content in UME curricula is the first step towards addressing these gaps. Prediabetes education in medical school should include content regarding clinical guidelines for screening and diagnosing prediabetes as well evidence-based recommendations for managing prediabetes. This content should be addressed in preclinical education, then reinforced and practiced in clinical experiences.

While there is an opportunity to improve students’ knowledge in all domains assessed in this study, the largest knowledge gaps were in clinical management of patients with prediabetes. Given the large volume of patients with prediabetes that the average medical student might be expected to encounter during their UME experience (roughly one-third of adults), there should be many opportunities to expose students to evidence-based management of patients with prediabetes, particularly during their primary care clerkships. However, the knowledge and practice gaps observed in practicing physicians highlight the need for associated faculty development on prediabetes management so that clinical preceptors reinforce these concepts, and to ensure that students can observe and participate in high quality preventive care during their clinical experiences. Faculty development coupled with updated prediabetes-related UME curricula are important steps that medical schools could consider helping address the growing type 2 diabetes epidemic.

A limitation of the study is that the medical students who completed the questionnaire are comprised of students attending the 2016 AMA HOD meeting who volunteered to participate. These students are AMA members and may not be a representative sample of US medical students. However, we are not aware of any evidence suggesting that the convenience sample of students who are members of the AMA would be more or less knowledgeable of preclinical and clinical management of prediabetes and diabetes prevention than medical students who are not AMA members. Next, the questionnaire response rate was 33% (197/596) when accounting for all medical students in attendance at the meeting. However, the total number of medical students attending the MSS sessions at times that questionnaires were administered was not measured, suggesting the actual response rate may be higher than reported. There also may be a positive response bias and overestimation of average knowledge levels if students who believed they knew more about the topic were more likely to complete the questionnaire. The results suggest there is little if any upward bias in knowledge due to these factors. Additionally, the questionnaire was not validated prior to administration to these students. The results from this phase of research can aid the development of a reliable and valid prediabetes knowledge test, similar to those developed and validated for diabetes [23].

## Conclusions

This study, using a questionnaire administered at the AMA’s 2016 HOD meeting, highlighted the low medical student performance on prediabetes knowledge. The average student answered fewer than half of the questionnaire questions correctly. Overall performance varied slightly by the students’ year in medical school, but only for certain questions between first- and second-year versus third- and fourth- year students. The results suggest a need for a review of current undergraduate medical school curricula, and for potential improvements to increase the awareness of screening for prediabetes as well the benefits of the LCPs that are part of the National DPP.

## Additional file

Additional file 1: Prediabetes Survey. (DOCX 50 kb)

## Data Availability

The datasets generated and analyzed during the current study are not available for public use and may be made available from the corresponding author on reasonable request.

## Abbreviations

AMA: American Medical Association
ANOVA: Analysis of Variance
BMI: Body mass index
CDC: Centers for Disease Control and Prevention
HbA1c: Hemoglobin A1c
HOD: House of Delegates
LCP: Lifestyle Change Program
MSS: Medical Student Section
National DPP: National Diabetes Prevention Program
UME: Undergraduate Medical Education
US: United States
USPSTF: United States Preventative Services Task Force
