# Supplementary material for: An assessment of medical students’ knowledge of prediabetes and diabetes prevention
Source: BMC Med Educ. 2019 Jul 29;19:285. doi: 10.1186/s12909-019-1721-9 (PMC6664721; doi:10.1186/s12909-019-1721-9)
Supplement: Supplementary file 1 — Prediabetes Survey. (DOCX 50 kb) [file 12909_2019_1721_MOESM1_ESM.docx]

Instructions: Please select one answer for each question.

1. What proportion of adults in the United States has prediabetes?

|  | 1 in 2 |
| --- | --- |
|  | 1 in 3 |
|  | 1 in 5 |
|  | 1 in 10 |

1. Which of the following is NOT a risk factor for prediabetes?

|  | Family history of type 2 diabetes mellitus |
| --- | --- |
|  | History of gestational diabetes |
|  | BMI of 20 |
|  | Asian race |

1. A patient with an A1Cof 6.5 has prediabetes.

|  | True |
| --- | --- |
|  | False |

1. The 2015 United States Preventive Services Task Force (USPSTF) recommendation statement about screening for abnormal glucose and type 2 diabetes mellitus recommends screening adults for abnormal glucose if they are:

|  | Overweight or obese at any age |
| --- | --- |
|  | Age 40 to 70 and overweight or obese |
|  | Over the age of 45 |
|  | Over the age of 45 and have at least one additional risk factor for abnormal glucose |

1. Which of the following best describes the abilities of metformin and the National Diabetes Prevention Program to reduce the incidence of type 2 diabetes mellitus among individuals with prediabetes?

|  | Neither metformin nor the National Diabetes Prevention Program are more effective than placebo |
| --- | --- |
|  | Both metformin and the National Diabetes Prevention Program are more effective than placebo, and they are similarly effective to each other |
|  | Metformin is nearly twice as effective as the National Diabetes Prevention Program (both are better than placebo) |
|  | The National Diabetes Prevention Program is nearly twice as effective as metformin (both are better than placebo) |

1. Individuals who participate in the National Diabetes Prevention Program can help prevent or delay the onset of type 2 diabetes if they lose a minimum of:

|  | 5 to 7 percent of their body weight |
| --- | --- |
|  | 10 to 12 percent of their body weight |
|  | 15 percent of their body weight |

Tell us who you are:

|  | Medical student  (Select year in medical school) | | | |
| --- | --- | --- | --- | --- |
|  | M1 | M2 | M3 | M4 |
|  | Resident | | | |
|  | Fellow | | | |
|  | Other  (Please specify)_________________ | | | |
